# Non-Invasive Histologic Markers of Liver Disease in Patients With Chronic Hepatitis B

**DOI:** 10.5812/hepatmon.14228

**Published:** 2014-02-28

**Authors:** Simin Dokht Shoaei, Shahnaz Sali, Mehdi Karamipour, Esmail Riahi

**Affiliations:** 1 Clinical Research and Development Center, Imam Hossein Hospital, Shahid Beheshti University of Medical Sciences, Tehran, IR Iran; 2 Infectious Diseases and Tropical Medicine Research Center, Shahid Beheshti University of Medical Sciences, Tehran, IR Iran; 3 Physiology Department, School of Medicine, Shahid Beheshti University of Medical Sciences, Tehran, IR Iran

**Keywords:** Chronic Hepatitis B, Liver Cirrhosis, Liver Disease

## Abstract

**Background:**

An exact histologic staging of liver fibrosis is essential for identifying the best therapeutic strategy and determining the disease prognosis in patients with chronic hepatitis B (CHB). While liver biopsy has a vital role in the management of liver diseases, it also sustains some limitations hampering its widespread use.

**Objectives:**

In this study, we evaluated and compared several available indices of the severity of liver diseases in patients with hepatitis.

**Patients and Methods:**

Exclusion criteria were as follows: decompensated liver disease, alcoholic liver disease or alcohol intake of 40 g or more per week; co-infection with human immunodeficiency virus, hepatitis C virus, or hepatitis D virus.

**Results:**

Results showed that AST to platelet ratio index (APRI) (odds ratio = 2.35, P = 0.01) and age (odds ratio = 1.04, P = 0.007) were independently predictive of the presence of significant liver necrosis and inflammation. On the other hand, AARPRI (odds ratio = 3.8, P = 0.07), age (odds ratio = 1.04, P = 0.02), and ALT levels (odds ratio = 1.01, P = 0.007) were predictive of a significant liver fibrosis. Further analysis with receiver-operating curve showed that none of these predictors had a fair diagnostic value (area under the curve < 70).

**Conclusions:**

The APRI had the highest sensitivity and specificity (64% and 71%, respectively) for prediction of the presence of liver disease. We suggest that APRI may be applicable for the detection of a severe liver disease.

## 1. Background

According to the World Health Organization, two billion people are infected with hepatitis B virus (HBV) worldwide; from which, 350 million and 40 million, have developed chronic hepatitis B (CHB) and chronic active hepatitis B with all their sequels, respectively (1). Chronic HBV infection commonly causes liver fibrosis which often progresses to liver cirrhosis and hepatocellular carcinoma (2, 3). Indeed, up to 40% of CHB patients would develop complications related to liver cirrhosis or hepatocellular carcinoma (4). Several clinical parameters, including male gender, older age, higher levels of alanine aminotransferase (ALT) and serum level of HBV DNA appear to be associated with the severity of liver disease (5, 6).

An exact histologic staging of liver fibrosis is essential for identifying the best therapeutic strategy and determining the disease prognosis in patients with chronic hepatitis B (CHB) (7). Percutaneous liver biopsy, the gold standard method in assessing the severity of liver disease, provides invaluable information regarding the necro-inflammation grade (NIA) and the stage of liver fibrosis (8, 9). While this procedure is fairly safe and simple, its use is hindered by several drawbacks. In addition, it is invasive and requires a hospital stay of 12-24 h. Moreover, procedure-related complications such as pain, bleeding, pneumothorax, bile-acid peritonitis or organ perforation might occur in up to 2% of patients (10, 11). There are also some concerns regarding the sampling error, variability in pathologic diagnosis, and reluctance of patients to undergo repeated biopsies for monitoring the disease progression (12-14). Consequently, there has been recent interest for developing non-invasive methods to detect the severity of liver disease. 

Several indices have been proposed as potential non-invasive predictors of the severity of liver fibrosis in CHB patients, including platelet count (15), hyaluronic acid (16), AST to platelet ratio index (APRI) (7, 16, 17), and Platelet/Age/Phosphatase/α-fetoprotein/AST index (PAPAS) (18). In this regard, some integrated models have been shown to consistently predict the level of liver fibrosis (1, 19, 20). In line with previous studies, we investigated the applicability of some of the commonly available non-invasive laboratory indices to the prediction of the level of liver NIA and fibrosis in CHB patients.

## 2. Objectives

This study was conducted in Labafinejad Hospital, Tehran, Iran from February 2011 to November 2012. The study protocol was approved by the Ethics Board of Shahid Beheshti University of Medical Sciences. A total of 137 patients with untreated CHB, with positive results for hepatitis B surface antigen (HBsAg) for at least six months, were recruited.

## 3. Patients and Methods

Exclusion criteria were as follows: decompensated liver disease, alcoholic liver disease or alcohol intake of 40 g or more per week and co-infection with human immunodeficiency virus, hepatitis C virus, or hepatitis D virus. Informed written consent was obtained from all patients prior to the enrollment. 

### 3.1. Laboratory Evaluation and Histological Examination

Patients’ demographic characteristics and laboratory parameters, including age, gender, hepatitis B e antigen (HBeAg) status, HBV DNA levels, ALT, AST, albumin, fasting blood sugar (FBS), triglyceride, cholesterol, hemoglobin, prothrombin time (PT), white blood cell count (WBC), and platelet count were recorded. All patients underwent percutaneous liver biopsy, through which liver parenchymal samples with at least 1.5 cm length were collected. Samples were then fixed in formalin, embedded in paraffin, sectioned into slices, and stained with hematoxylin and eosin for grading hepatic NIA (A0-A18) using the Knodell histologic activity index (21, 22) or collagen stain for staging the hepatic fibrosis (F0-F6) based on the Ishak fibrosis score (23). NIA grades were divided into three categories: normal to mild (A0-A6), moderate (A7-A12), and severe (A13-A18). In addition, the extent of liver fibrosis was categorized as: normal to mild (F0-F2), moderate (F3-F4), and severe (F5-F6).

### 3.2. Non-Invasive Predictors of Liver NIA and Fibrosis

Several potential noninvasive indices were evaluated for grading the liver inflammatory activity and the extent of liver fibrosis. These markers included the AST/ALT ratio (AAR), the AAR to platelet ratio index (AARPRI), the AST to platelet ratio index (APRI), and the age to platelet index (API). The APRI was calculated as AST/upper limit of normal (ULN)]/platelet count (×109/L) × 100. The ULN for AST was 30 IU/L. The AARPRI was calculated as AAR/ [platelet count (×109/L)/150]. The API was calculated as the sum of age and platelet count scores as follows: age (years) < 30 = 0, 30-39 = 1, 40-49 = 2, 50-59 = 3, 60-69 = 4, ≥ 70 = 5 and platelet count (×109/L) ≥ 225 = 0, 200-224 = 1, 175-199 = 2, 150-174 = 3, 125-149 = 4, ≤ 125 = 5 (15).

### 3.3. Statistical Analysis

All statistical analysis were performed using SPSS version 19.0 software. Continuous data were presented as the mean ± SD. For statistical analyses, the moderate and severe NIA and fibrosis were recoded as significant NIA and fibrosis, respectively. Therefore, the study subjects were divided into two groups based on the severity of NIA or fibrosis. For the NIA data, these categories included patients with no or mild NIA (grade A0-A6) and patients with significant NIA (grade A7-A18). For the liver fibrosis data, patients with stages F0 to F2 were categorized as no or mild fibrosis group, and patients with stages F3 to F6 were considered as the significant fibrosis group. The independent sample student’s t-test and the chi-square test were used to compare inter-group differences regarding the continuous and categorical variables, respectively. The primary endpoint was to determine any association between the histologic severity of liver disease and the paraclinical parameters assessed in this study. Spearman correlation coefficient was used to evaluate the correlation between each non-invasive marker and the degree of NIA or fibrosis. Stepwise backward logistic regression was used to assess the predictive value of each marker. The accuracy of each marker to predict the hepatic disease was assessed by the area under the receiver–operating characteristic (ROC) curves. The cut-off value was determined as the point where the sum of sensitivity and specificity values was the greatest. P values less than 0.05 were considered to be statistically significant.

## 4. Results

### 4.1. Patients’ Demographic Characteristics

In total, 137 patients with HBsAg-positive CHB were included in this study. Of these, 99 patients (72.3%) were men and 38 (27.7%) were women. The mean age (± standard deviation) was 40.13 ± 12.46 years. The detailed demographic and laboratory parameters of the study patients are listed in Table 1. Seventy-six patients had no or mild hepatic NIA (grades A0-A6) and 61 patients had significant NIA (grades A7-A18). Comparison of patients with no or mild NIA and patients with significant NIA showed that patients in the latter group were of older age, and had higher ALT and AST levels. However, HBeAg prevalence was lower in patients with significant NIA. Likewise, 77 patients had no or mild liver fibrosis (stages F0-F2) and 60 patients had significant fibrosis (stages F3-F6). Compared to the patients with no or mild liver fibrosis, patients with significant fibrosis were significantly older and had higher levels of FBS, AST, and ALT.

**Table 1.** Basic Clinical Characteristics and Laboratory Parameters of Study Subjects ^a^

Parameter	Total Cohort	Severity of NIA		P value	Severity of Fibrosis		P value
		No or Mild NIA	Significant NIA		No or Mild Fibrosis	Significant Fibrosis	
**Number of Patients**	137	76 (55.5)	61 (44.5%)	-	77 (56.2)	60 (43.8)	-
**Age, y**	40.13 ± 12.46	37.03 ± 11.22	44.00 ± 12.93	0.001	37.51 ± 11.39	43.52 ± 13.05	0.005
**Gender, Male**	99 (72.3)	58 (58.6)	41 (41.4)	0.25	55 (55.6)	44 (44.4)	0.84
**BMI** ^**b**^ **, kg/m** ^**2**^	24.59 ± 4.87	24.06 ± 4.57	25.28 ± 5.18	0.15	24.00 ± 4.70	25.35 ± 5.01	0.11
**HBeAg** ^**b **^ **Positive Disease **	51 (50)	35 (68.6)	16 (31.4)	0.01	33 (64.7)	18 (35.3)	0.16
**HBV DNA, IU/m**L	9452 ± 57362	14311 ± 78053	4107 ± 15992	0.48	509 ± 1086	18116 ± 80139	0.22
**WBC**^**b**^**, ×10**^**9**^**/**L	6.20 ± 1.73	6.17 ± 1.66	6.23 ± 1.82	0.83	6.33 ± 1.84	6.04 ± 1.57	0.33
**Platelet, ×10**^**9**^**/**L	203.96 ± 51.80	210.42 ± 45.99	195.91 ± 57.61	0.10	210.58 ± 48.11	195.47 ± 55.44	0.09
**Hemoglobin, g/**L	148.2 ± 17.6	147.72 ± 17.25	148.75 ± 18.22	0.73	148.84 ± 16.43	147.33 ± 19.18	0.62
**PT** ^**b**^ **, s**	13.15 ± 0.85	13.06 ± 0.86	13.26 ± 0.83	0.17	13.10 ± 0.84	13.23 ± 0.86	0.37
**FBS, mg/d**L	90.27 ± 13.8	88.98 ± 10.75	91.88 ± 16.80	0.22	88.09 ± 9.49	93.08 ± 17.57	0.05
**Triglyceride, mg/d**L	124.11 ± 63.65	124.02 ± 59.90	124.23 ± 68.54	0.98	117.26 ± 58.92	132.92 ± 68.75	0.15
**Cholesterol, mg/d**L	169.84 ± 41.73	164.64 ± 36.76	176.33 ± 46.71	0.10	168.90 ± 38.75	171.07 ± 45.58	0.76
**Albumin, g/m**L	4.11 ± 0.54	4.15 ± 0.47	4.08 ± 0.62	0.44	4.19 ± 0.44	4.03 ± 0.63	0.09
**Total Protein, g/**L	74.84 ± 5.88	7.49 ± 0.46	7.47 ± 0.72	0.85	7.47 ± 0.49	7.50 ± 0.69	0.77
**AST**^**b**^**, IU/**L	71.47 ± 102.66	50.33 ± 24.81	97.82 ± 147.82	0.01	50.53 ± 24.10	98.35 ± 149.13	0.01
**ALT**^**b**^**, IU/**L	105.1 ± 146.54	76.09 ± 45.80	141.23 ± 208.95	0.02	74.06 ± 39.80	144.92 ± 211.16	0.01

^a^ Data are presented as mean ± SD. Gender and HBeAg-positive disease are expressed as frequency and percentage.

^b^ Abbreviations: ALT, alanine aminotransferase; AST, aspartate aminotransferase; BMI, body mass index; HBeAg, hepatitis B e antigen; PT, prothrombin time; WBC, white blood cell.

### 4.2. Non-Invasive Indices of Prediction of Liver Disease

Routine non-invasive indices of liver disease, including AAR, AARPRI, APRI, and API were measured and analyzed regarding their relation with the severity of liver NIA and fibrosis. Independent sample t-test indicated that APRI and API were significantly higher in patients with significant liver disease (NIA and fibrosis) compared to those with no or mild liver disease (Table 2). 

Moreover, bivariate Spearman’s correlation test showed that there were significant correlations between age, AST, ALT, AARPRI, APRI, and API and the severity of liver NIA (A0-A18) and fibrosis (F0-F6, Table 3). To identify the independent predictors of liver disease, multiple logistic regression test was performed using the variables showing statistically significant differences in the univariate and bivariate analyses of the measured factors, including AARPRI, APRI, API, age, AST, and ALT. Of these, only APRI (odds ratio = 2.35, P = 0.01) and age (odds ratio = 1.04, P = 0.007) were found to be independently indicative of the presence of significant liver NIA. On the other hand, AARPRI (odds ratio = 3.8, P = 0.07), age (odds ratio = 1.04, P = 0.02), and ALT levels (odds ratio = 1.01, P = 0.007) were predictive of a significant liver fibrosis. To assess the accuracy of various variables for prediction of liver disease, the ROC curve was constructed for each variable and the area under the ROC curve (AUROC) was used to determine the predictive accuracy of each parameter. Results (Tables 4 and 5) showed that the AARPRI failed to accurately predict the severity of liver NIA and fibrosis (AUROC < 60), and the predictive values of other variables including APRI, API, AST, ALT, and age were poor (60 < AUROC < 70). None of the predictors had a good or powerful diagnostic value. However, APRI had the best sensitivity and specificity (64% and 71%, respectively) for prediction of liver disease.

**Table 2.** Non-Invasive Indices of Predicting the Severity of Liver NIA

Non-invasive indices	Severity of Liver NIA, Mean ± SD			Severity of Liver Fibrosis, Mean ± SD		
	No or Mild	Significant	P value	No or Mild	Significant	P value
**AAR** ^**a**^	0.78 ± 0.37	0.73 ± 0.25	0.38	0.76 ± 0.33	0.75 ± 0.31	0.79
**AARPRI** ^**a**^	0.58 ± 0.28	0.61 ± 0.30	0.42	0.57 ± 0.27	0.62 ± 0.31	0.28
**APRI** ^**a**^	0.83 ± 0.47	1.93 ± 3.15	0.009	0.85 ± 0.50	1.92 ± 3.18	0.01
**API** ^**a**^	2.59 ± 1.85	3.92 ± 2.31	0.000	2.70 ± 1.87	3.80 ± 2.36	0.004

^a^ AAR, AST/ALT; AARPRI, AAR to platelet ratio index; API, age to platelet index; APRI, AST to platelet ratio index.

**Table 3.** Correlation Between the Potential Predictors of Liver Disease and Severity of Liver NIA and Fibrosis

Non-Invasive Indices	Correlation With Severity of Liver NIA (rho Coefficient)	P value	Correlation With Severity of Liver Fibrosis (rho Coefficient)	P value
**Age, y**	0.23	0.01	0.31	0.001
**AST** ^**a**^	0.31	0.001	0.30	0.001
**ALT** ^**a**^	0.22	0.02	0.22	0.02
**AAR** ^**b**^	0.10	0.31	0.09	0.34
**AARPRI ** ^**b**^	0.25	0.008	0.24	0.009
**APRI ** ^**b**^	0.37	0.000	0.38	0.000
**API ** ^**b**^	0.36	0.000	0.31	0.001

^a^ Abbreviations: ALT, alanine aminotransferase; AST, aspartate aminotransferase.

^b^ AAR, AST/ALT; AARPRI, AAR to platelet ratio index; API, age to platelet index; APRI, AST to platelet ratio index.

**Table 4.** The AUROC, Cut-Off Value, Sensitivity and Specificity of Variables in Relation to Significant Liver NIA

Variable	AUROC	P value	Cut-off	Sensitivity	Specificity
**AARPRI** ^**a**^	0.55	0.31	0.60	0.49	0.66
**APRI ** ^**a**^	0.66	0.04	0.85	0.64	0.71
**API ** ^**a**^	0.66	0.04	4.50	0.39	0.87
**AST**^** a,b**^**, IU/**L	0.65	0.04	52.50	0.57	0.71
**ALT **^**b**^**, IU/**L	0.62	0.04	63.50	0.72	0.51
**Age, y**	0.67	0.04	49.50	0.39	0.92

^a^ AARPRI, AAR to platelet ratio index; API, age to platelet index; APRI, AST to platelet ratio index.

^b^ Abbreviations: ALT, alanine aminotransferase; AST, aspartate aminotransferase.

**Table 5.** The AUROC, Cut-Off Value, Sensitivity and Specificity of Variables in Relation to Significant Liver Fibrosis

Variable	AUROC	P value	Cut-off	Sensitivity	Specificity
**AARPRI** ^**a**^	0.55	0.26	0.50	0.63	0.52
**APRI** ^**a**^	0.65	0.002	0.85	0.65	0.71
**API** ^** a**^	0.64	0.006	2.50	0.72	0.51
**AST**^**a, b**^**, IU/**L	0.63	0.01	52.50	0.58	0.71
**ALT**^**b**^**, IU/**L	0.62	0.01	81.50	0.58	0.71
**Age**	0.65	0.003	43.50	0.60	0.73

^a^ AARPRI, AAR to platelet ratio index; API, age to platelet index; APRI, AST to platelet ratio index.

^b^ Abbreviations: ALT, alanine aminotransferase; AST, aspartate aminotransferase.

## 5. Discussion

Several studies have investigated the potential usefulness of serum biomarkers of liver function and indices derived from these biomarkers in prediction of liver fibrosis and cirrhosis in patients with hepatitis C and B (7, 17, 19, 24). This is due to limitations associated with the gold standard liver biopsy for long-term monitoring of the liver disease severity. While liver biopsy is often indispensable in the management of patients with liver disease, physicians and patients might be reluctant to do this due to its associated risks (11). The most frequently reported complications of liver biopsy are pain (occurring in up to 84% of patients), bleeding (the most important complication), and death (typically related to hemorrhage) (11). Consequently, there is currently a high level of interest in non-invasive markers able to provide accurate information about the degree of liver fibrosis and necrosis in patients with chronic liver diseases (25).

In the present study, we evaluated several non-invasive markers previously reported to be indicative of the severity of liver disease based on a number of readily available laboratory tests. These include platelet count, HBV DNA load, HBeAg, AST, ALT, AAR, AARPRI, APRI and API. Results obtained from our study showed that while AST, ALT, AARPRI, APRI and API, as well as the variable age were significantly associated with the severity of hepatic histological disease, only APRI and age were independently predictive of significant (moderate to severe) liver NIA. Moreover, AARPRI, age, and ALT appeared to be predictive of a significant liver fibrosis. APRI and AARPRI were more closely related to the severe liver NIA and fibrosis, respectively as they had the highest odds ratios in the statistical analysis. However, AARPRI cannot be a potential diagnostic marker for liver disease since it had a very low accuracy. Nevertheless, APRI had the best sensitivity and specificity, and thus the highest accuracy for predicting a liver disease. The area under the ROC curve for APRI was found to be 0.66. This is similar to other studies that collectively reported an AUROC for APRI of between 0.63 and 71 for prediction of liver fibrosis (17, 18, 26). Our results suggest that the accuracy of APRI for prediction of significant liver disease is relatively fair. Other studies also suggested that APRI had limited value to identify hepatitis B-related significant fibrosis and cirrhosis (7, 17, 26); although another study reported that APRI could identify significant and extensive fibrosis with an AUROC value of 0.72 (1, 20, 24). It has been reported that the API is able to predict significant liver fibrosis with an AUROC of 0.70 to 0.81 (1, 20, 24). However, another study reported that API had poor reliability for prediction of liver cirrhosis (AUROC ~ 0.61) (15). In the present study, the AUROC for API was 0.64 to detect liver fibrosis, and it failed to independently predict a significant liver NIA or fibrosis. Therefore, it seems that this index may not be a reliable marker for the prediction of the severity of liver disease. In conclusion, our study showed that while the APRI displayed relatively fair accuracy for the detection of a severe liver disease, other non-invasive indices evaluated in this study were not reliable predictors of the severity of liver disease.
